# Supplementary figures and images for: Creation of novel alleles of fragrance gene OsBADH2 in rice through CRISPR/Cas9 mediated gene editing
Source: PLoS One. 2020 Aug 12;15(8):e0237018. doi: 10.1371/journal.pone.0237018 (PMC7423090; doi:10.1371/journal.pone.0237018)

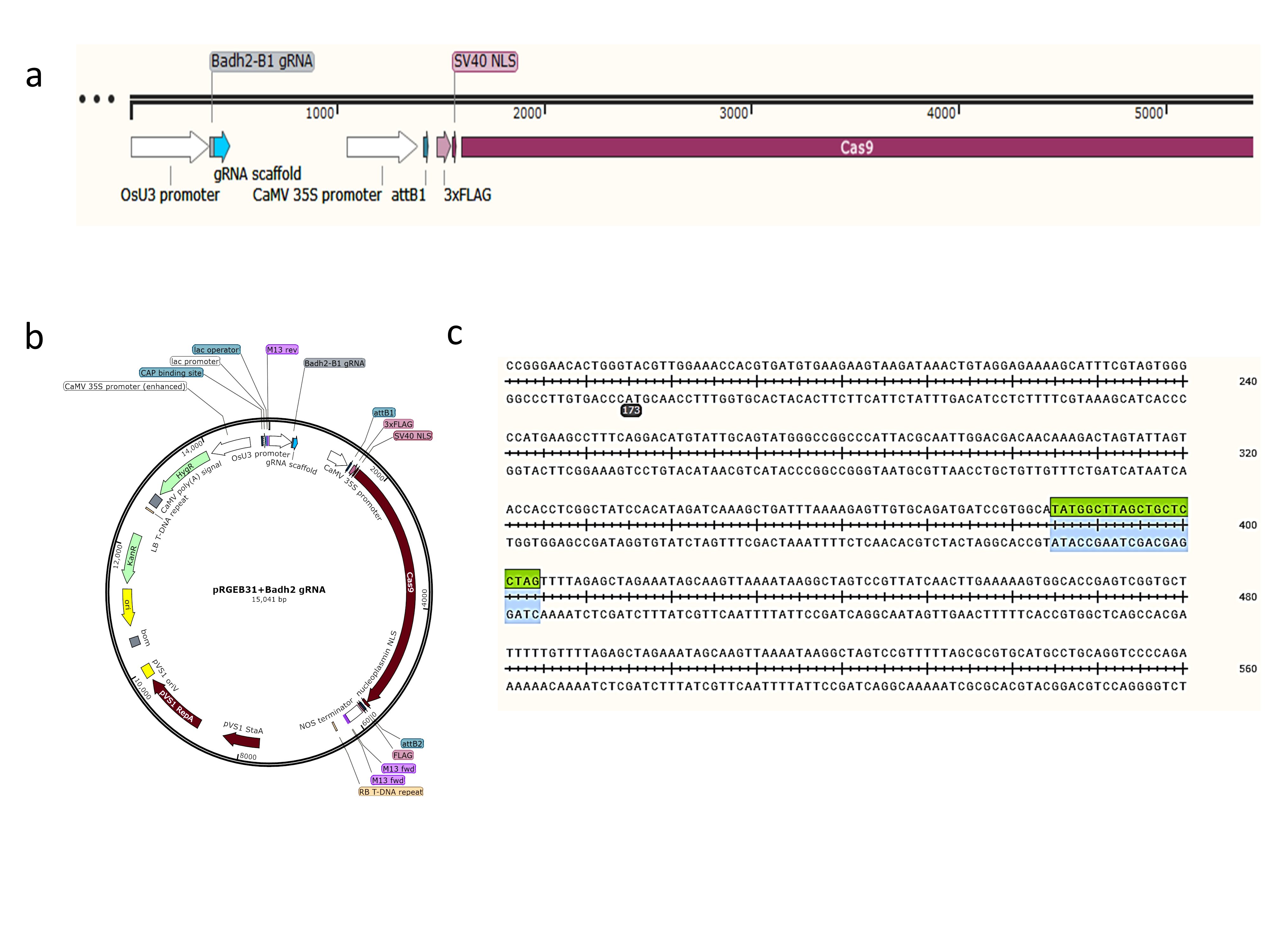

Supplement: S1 Fig — a) pRGEB31 possessing OsBadh2 guide RNA b) Sequence analysis showing the presence of OsBADH2-sgRNA in pRGEB31. (TIF) [file pone.0237018.s001.tif]

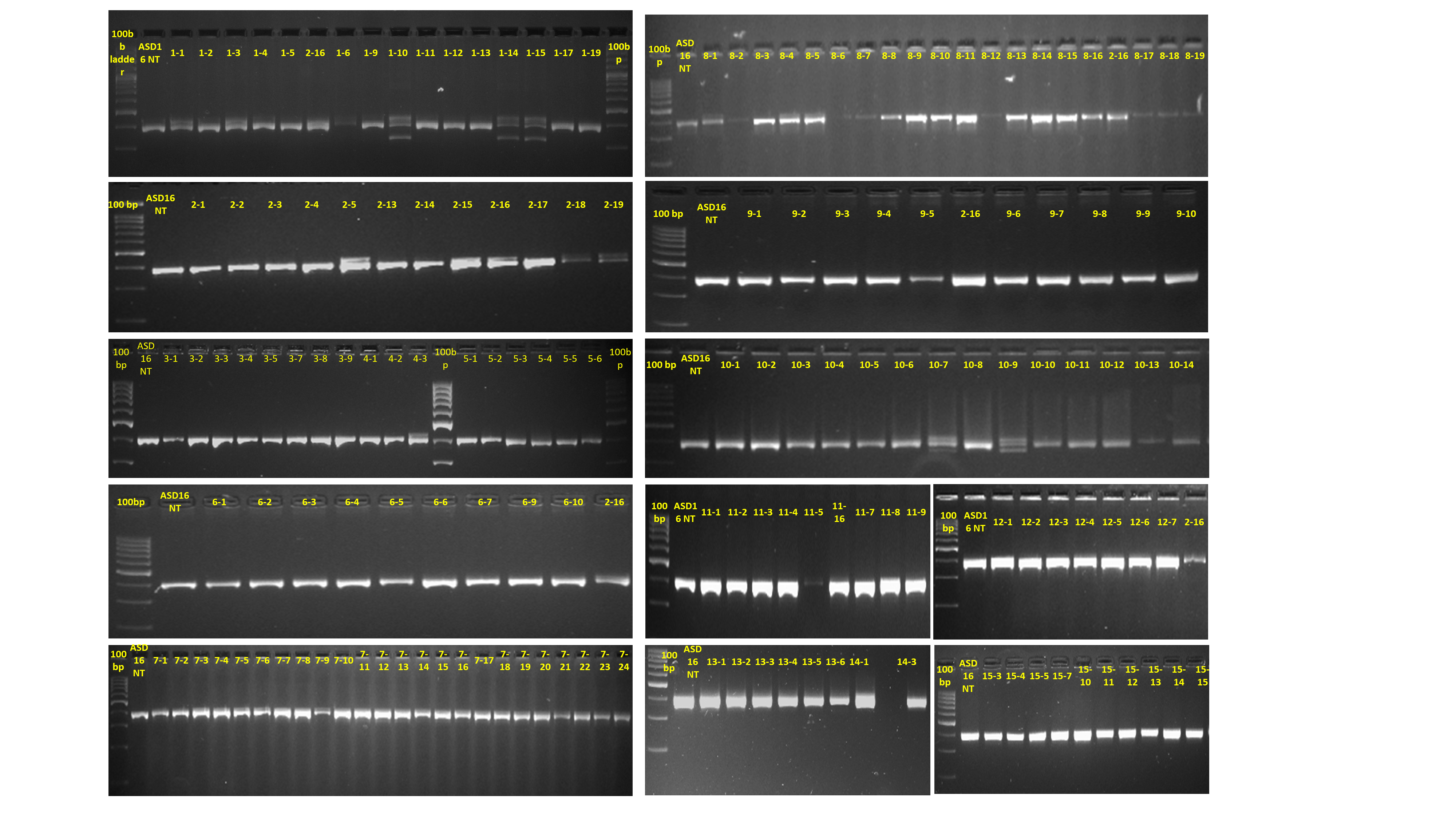

Supplement: S2 Fig — (TIF) [file pone.0237018.s002.tif]

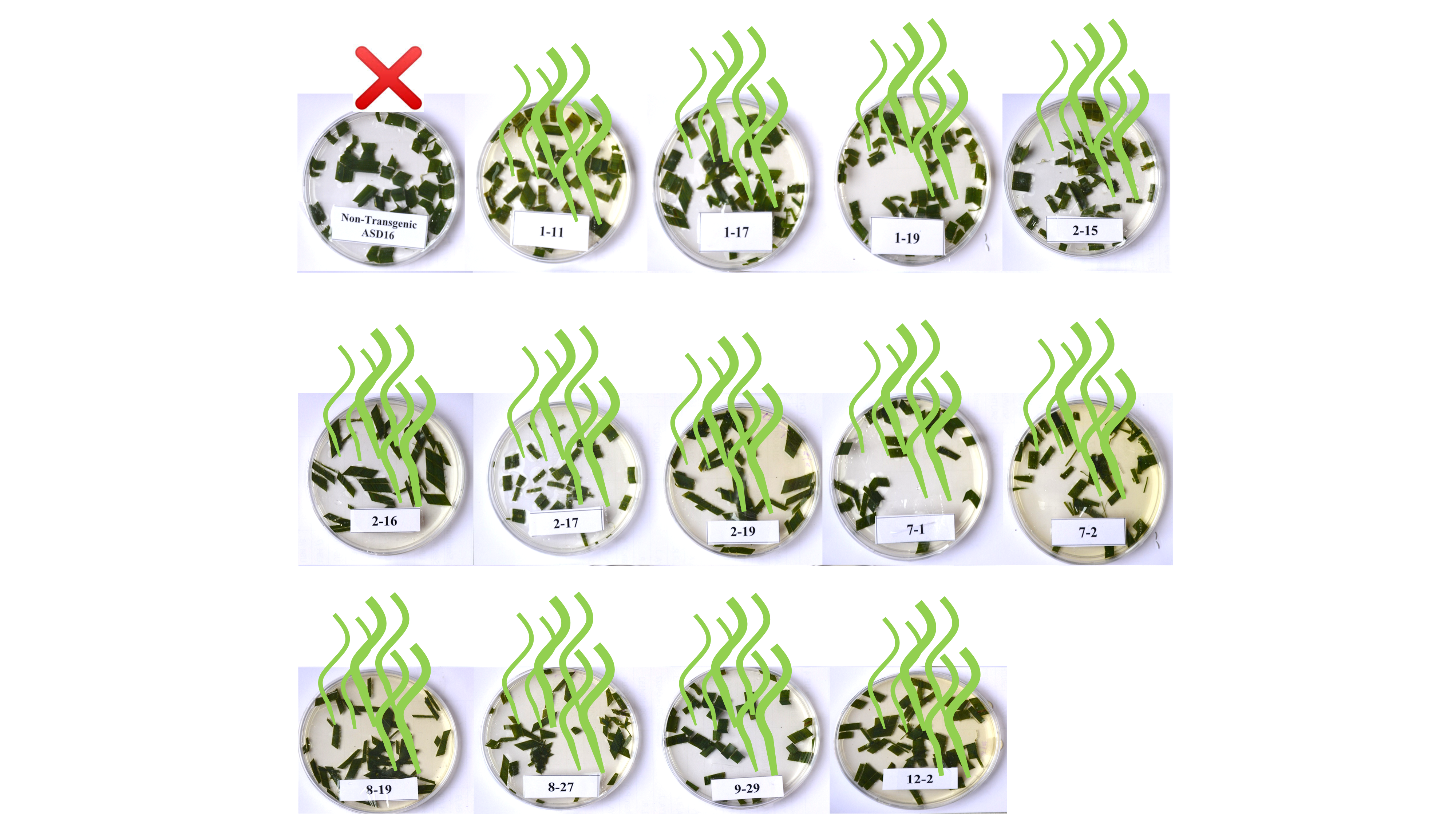

Supplement: S3 Fig — (TIF) [file pone.0237018.s003.tif]

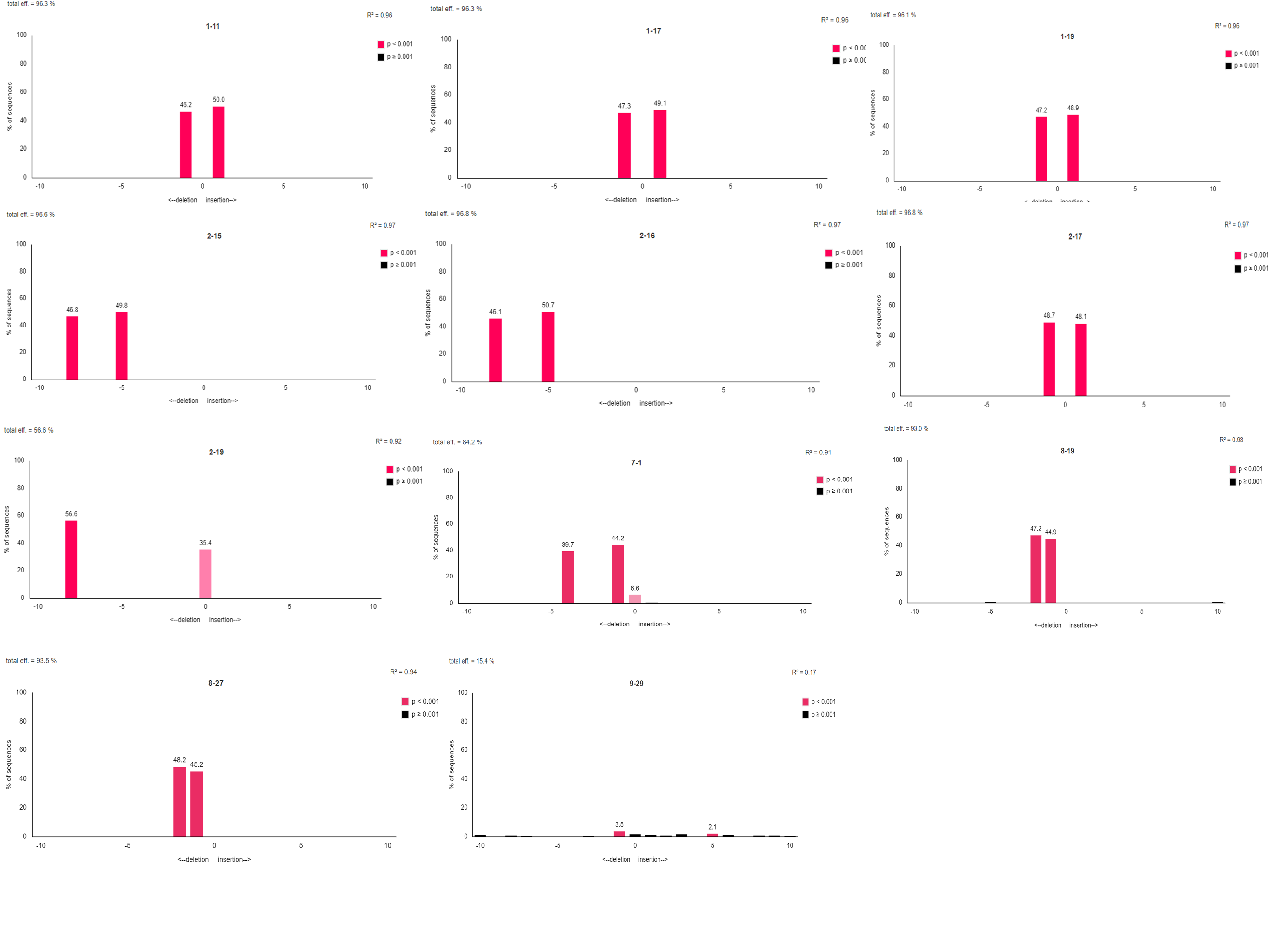

Supplement: S4 Fig — Prediction of putative bi-allelic mutants using TIDE analysis. (TIF) [file pone.0237018.s004.tif]

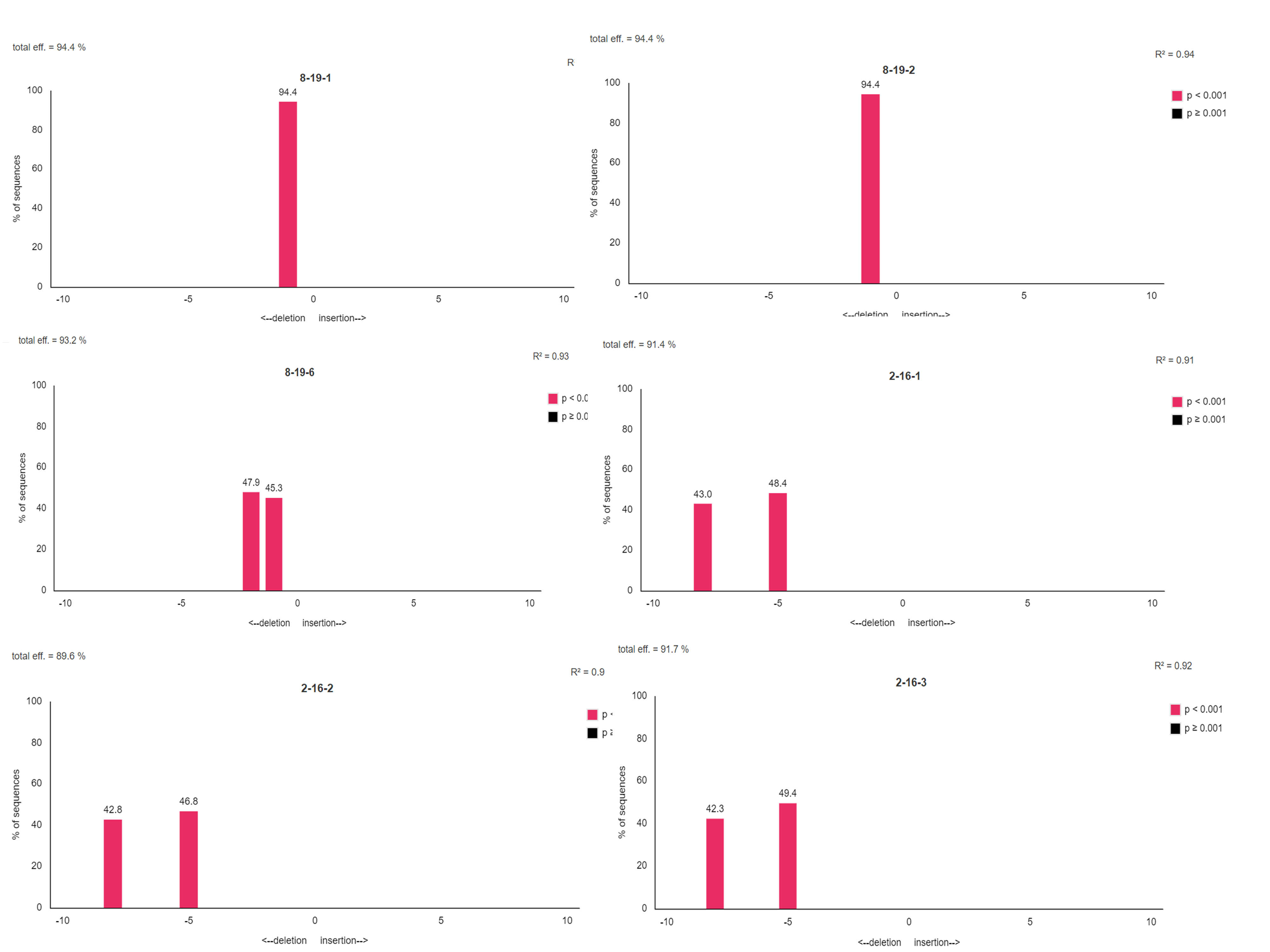

Supplement: S5 Fig — (TIF) [file pone.0237018.s005.tif]

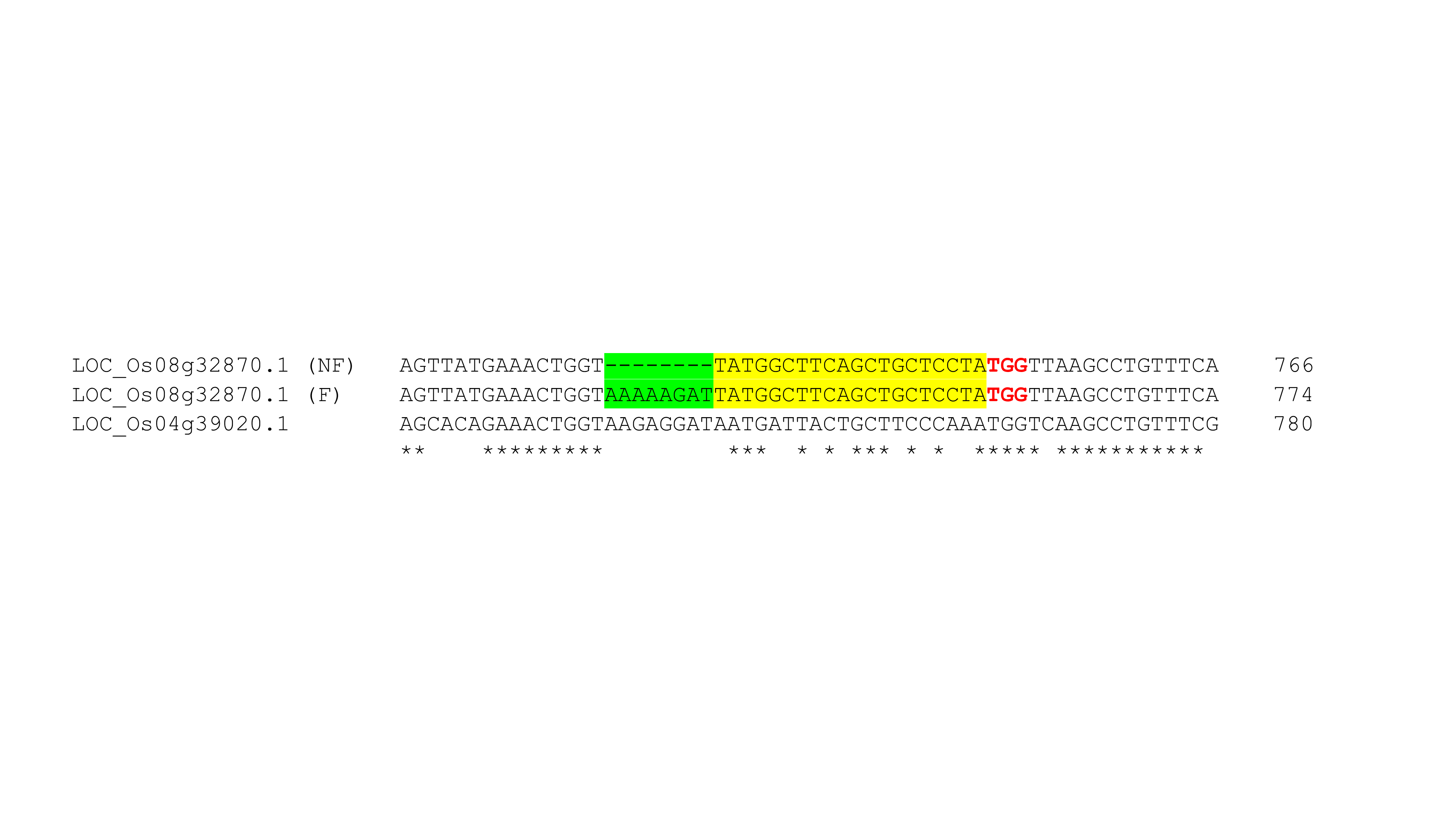

Supplement: S6 Fig — (TIF) [file pone.0237018.s006.tif]
