# Supplementary material for: Creation of novel alleles of fragrance gene OsBADH2 in rice through CRISPR/Cas9 mediated gene editing
Source: PLoS One. 2020 Aug 12;15(8):e0237018. doi: 10.1371/journal.pone.0237018 (PMC7423090; doi:10.1371/journal.pone.0237018)
Supplement: S1 Raw images — (PDF) [file pone.0237018.s008.pdf]

Raw image for Figure 2j

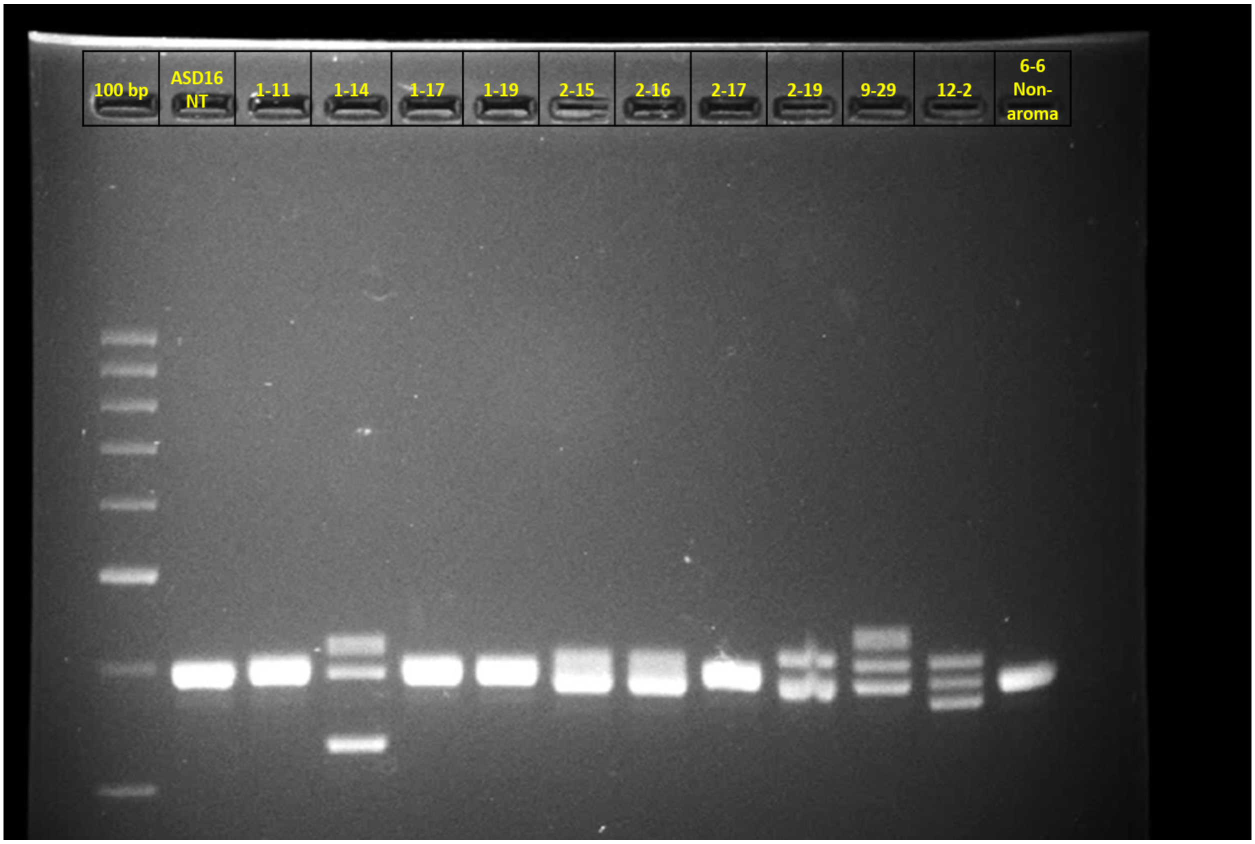

Raw Image for S2

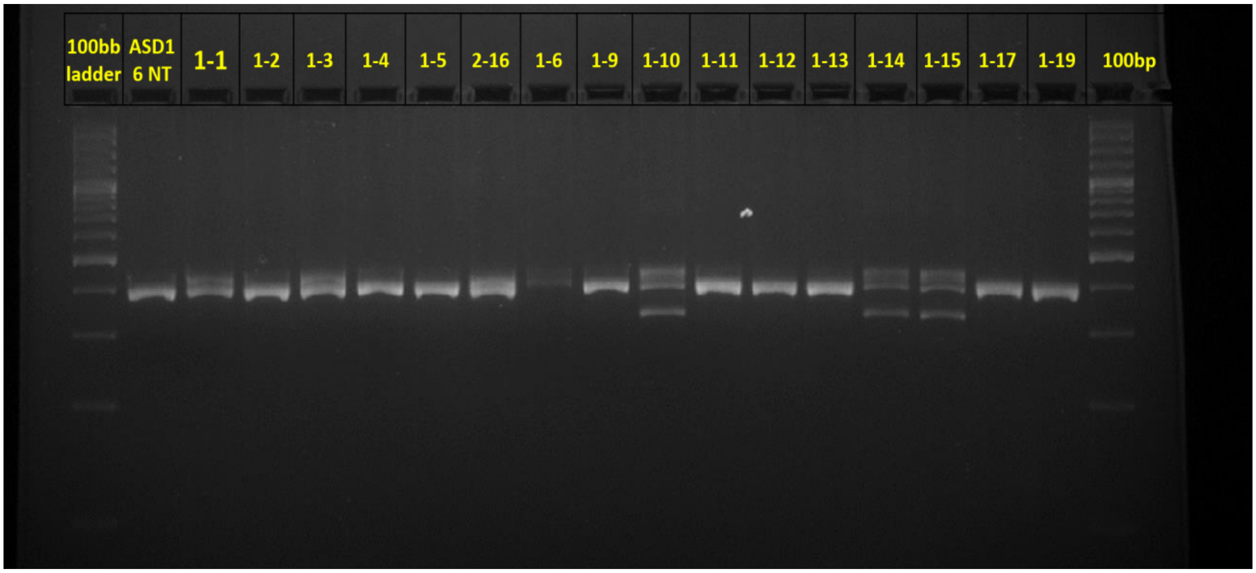

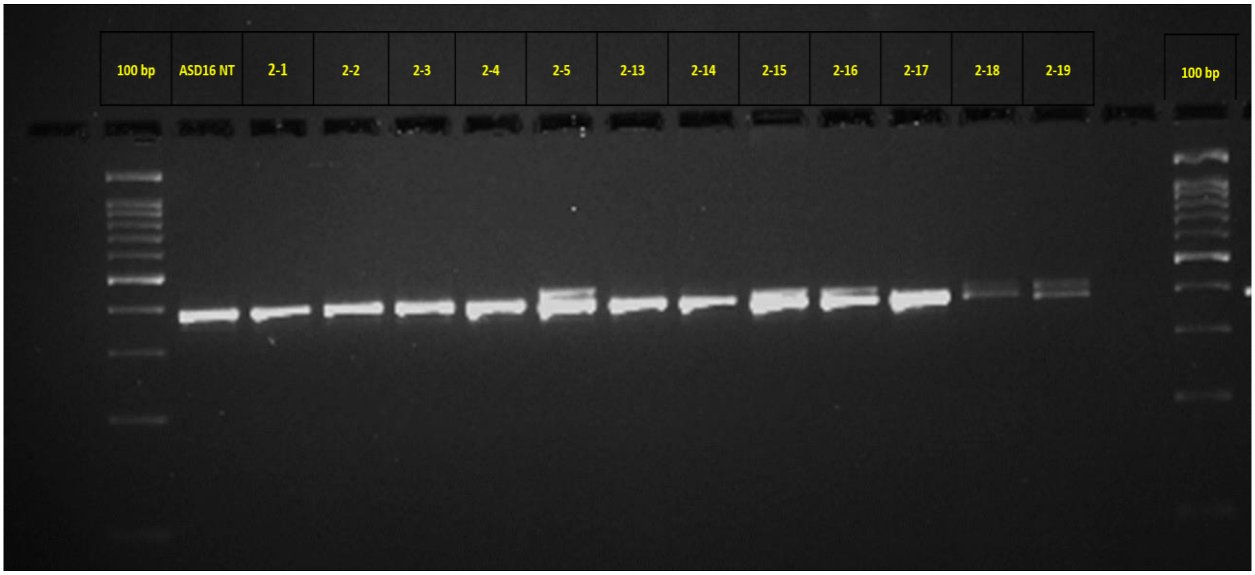

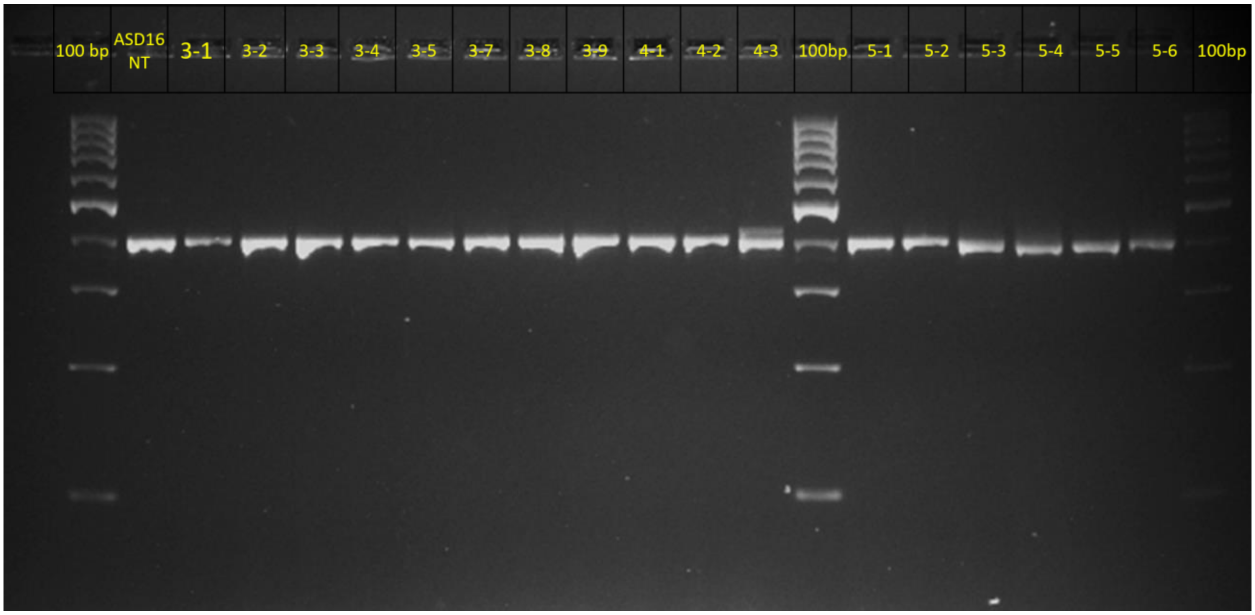

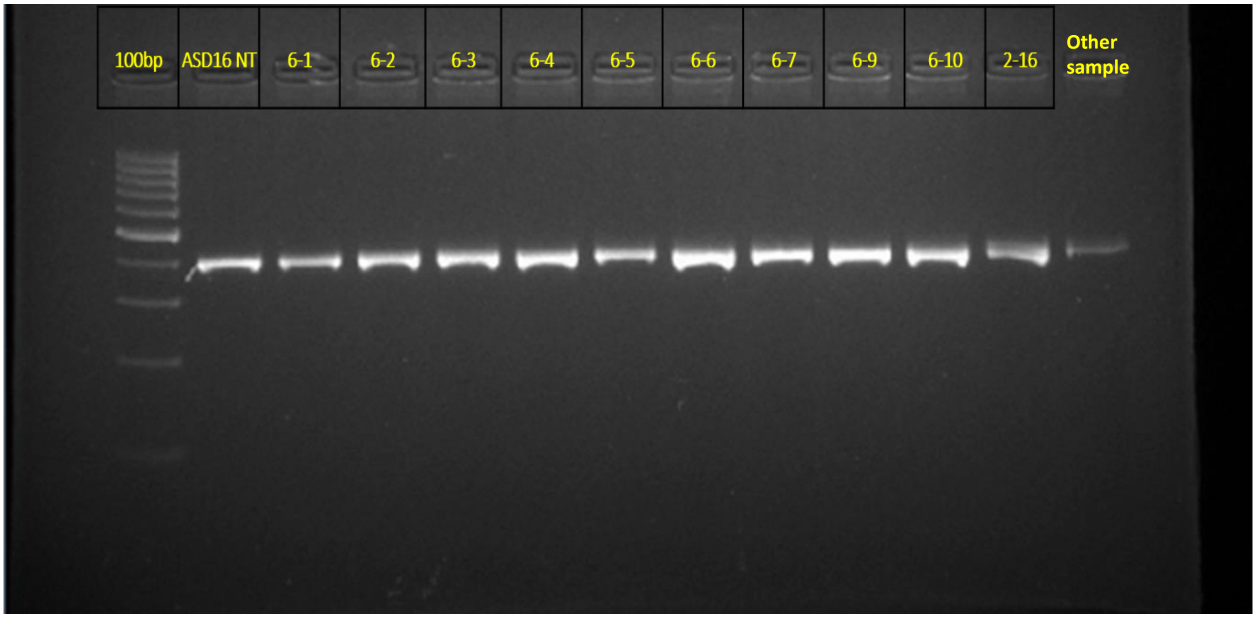
100bp
ASD16 NT
6-1
6-2
6-3
6-4
6-5
6-6
6-7
6-9
6-10
2-16
Other sample

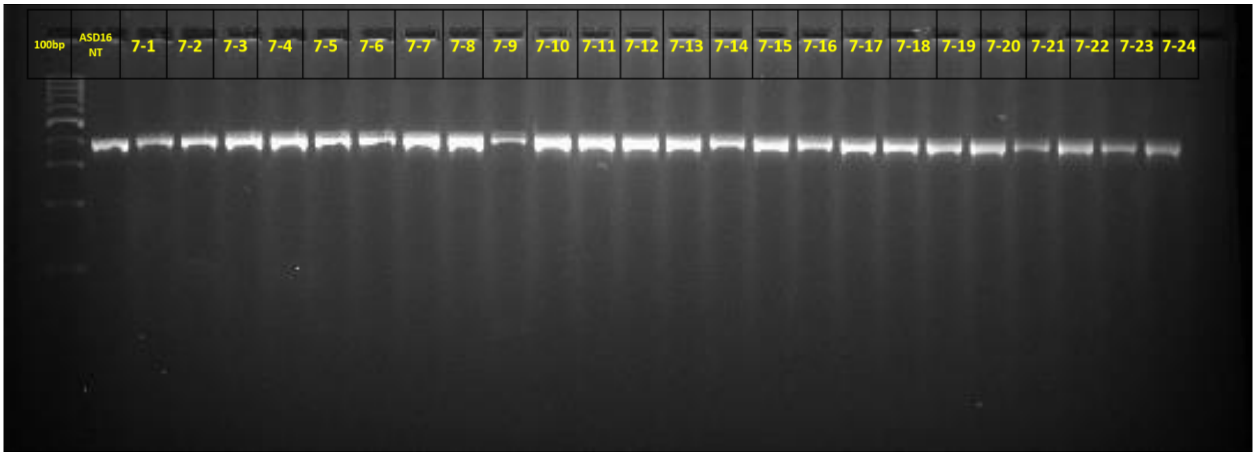
100bp
ASD16 NT
7-1
7-2
7-3
7-4
7-5
7-6
7-7
7-8
7-9
7-10
7-11
7-12
7-13
7-14
7-15
7-16
7-17
7-18
7-19
7-20
7-21
7-22
7-23
7-24

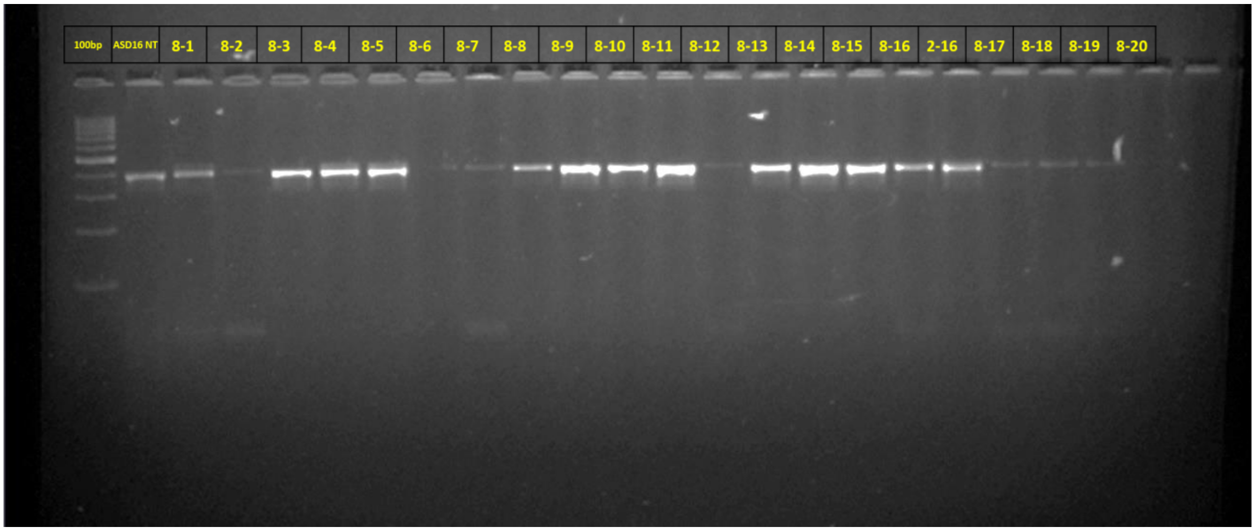
100bp
ASD16 NT
8-1
8-2
8-3
8-4
8-5
8-6
8-7
8-8
8-9
8-10
8-11
8-12
8-13
8-14
8-15
8-16
2-16
8-17
8-18
8-19
8-20

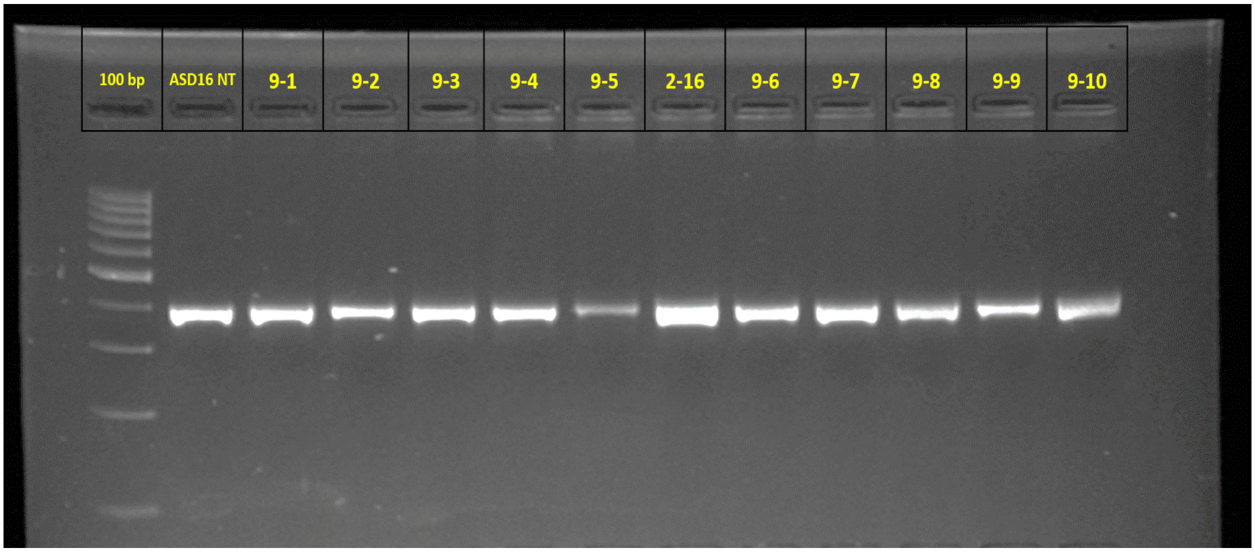
100 bp
ASD16 NT
9-1
9-2
9-3
9-4
9-5
2-16
9-6
9-7
9-8
9-9
9-10

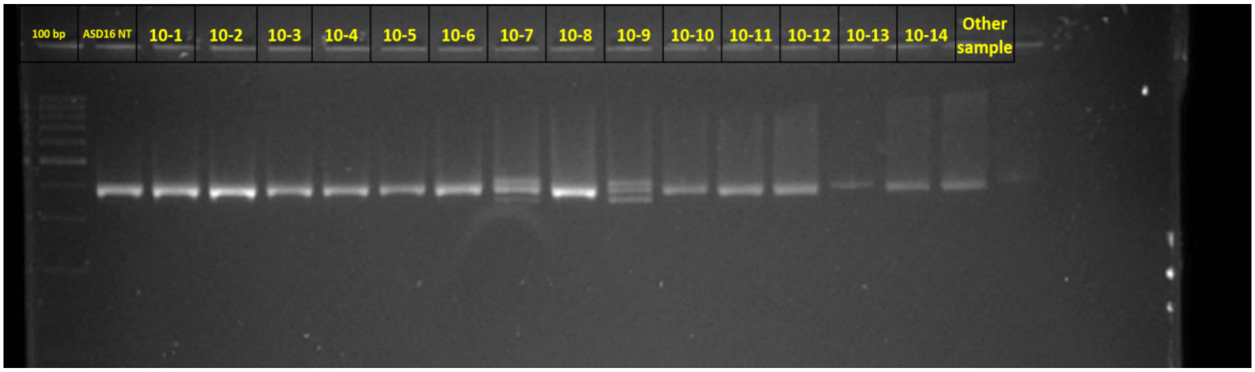
100 bp
ASD16 NT
10-1
10-2
10-3
10-4
10-5
10-6
10-7
10-8
10-9
10-10
10-11
10-12
10-13
10-14
Other sample

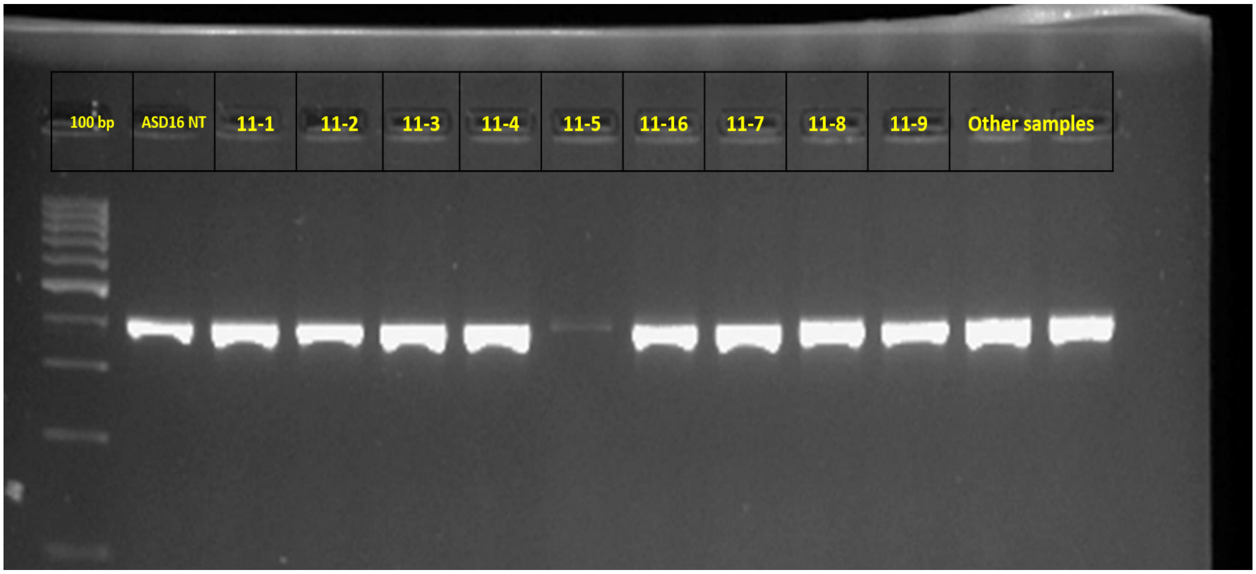
100 bp
ASD16 NT
11-1
11-2
11-3
11-4
11-5
11-16
11-7
11-8
11-9
Other samples

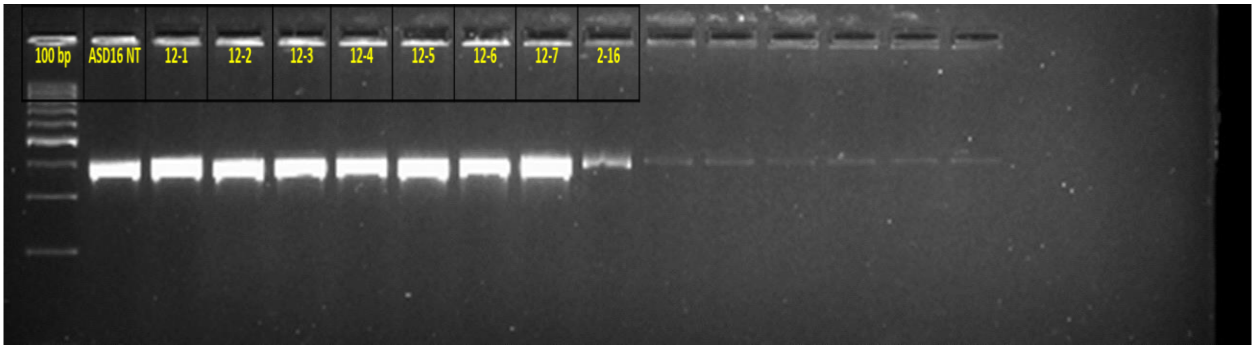
100 bp
ASD16 NT
12-1
12-2
12-3
12-4
12-5
12-6
12-7
2-16

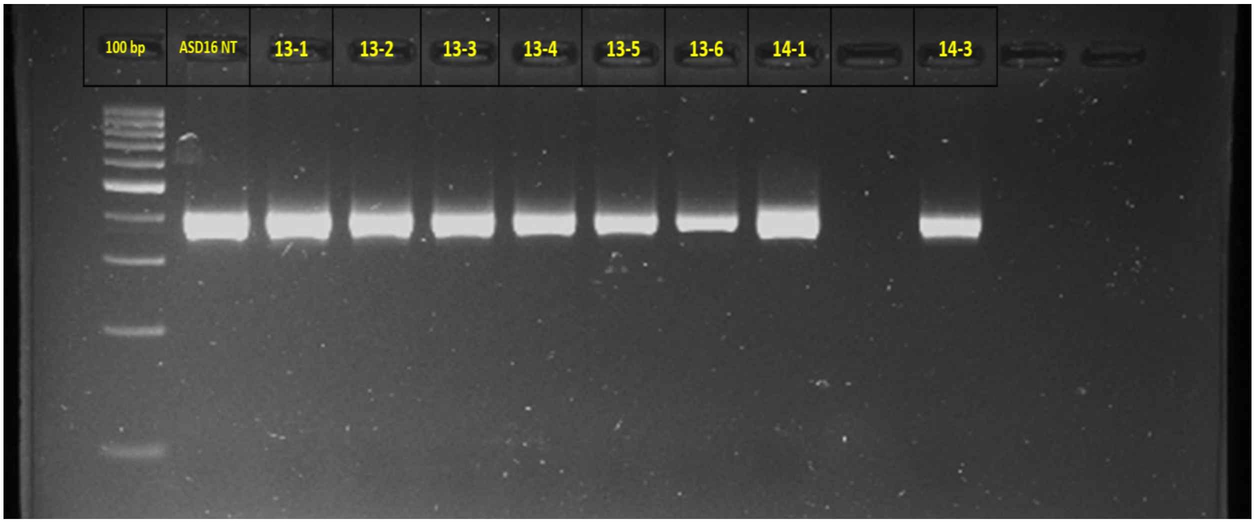
100 bp
ASD16 NT
13-1
13-2
13-3
13-4
13-5
13-6
14-1
14-3

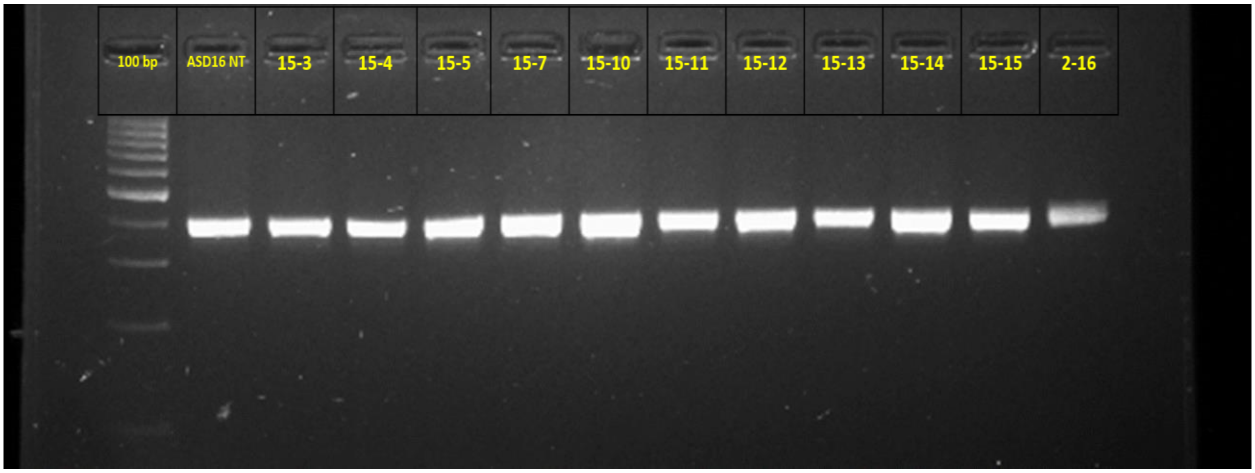
100 bp
ASD16 NT
15-3
15-4
15-5
15-7
15-10
15-11
15-12
15-13
15-14
15-15
2-16

S6 Raw image

```
CLUSTAL O(1.2.4) multiple sequence alignment


LOC_Os08g32870.1_NF      ------ATGGCCACGGCGATCCCGCAGCGGCAGCTCTTCGTCGCCGGCGAGTGGCGCGCC 54
LOC_Os08g32870.1_F       ------ATGGCCACGGCGATCCCGCAGCGGCAGCTCTTCGTCGCCGGCGAGTGGCGCGCC 54
LOC_Os04g39020.1         ATGGCCGCGCCGTCGGCGATCCCCCGCCGCGGCCTGTTCATCGGCGGCGGGTGGCGGGAG 60
                                 * *  ********** *  **    ** *** *** ***** ****** *

LOC_Os08g32870.1_NF      CCCGCGCTCGGCCGCCGCCTCCCCGTCGTCAACCCCGCCACCGAGTCCCCCATCGGCGAG 114
LOC_Os08g32870.1_F       CCCGCGCTCGGCCGCCGCCTCCCCGTCGTCAACCCCGCCACCGAGTCCCCCATCGGCGAG 114
LOC_Os04g39020.1         CCGTCCCTCGGCCGCCGCCTCCCCGTCGTCAACCCGGCCACGGAGGCAACCATCGGTGAC 120
                         **  * ***************************** ***** *** *  ******* **

LOC_Os08g32870.1_NF      ATCCCGGCGGGCACGGCGGAGGACGTGGACGCGGCGGTGGCGGCGGCGCGGGAGGCGCTG 174
LOC_Os08g32870.1_F       ATCCCGGCGGGCACGGCGGAGGACGTGGACGCGGCGGTGGCGGCGGCGCGGGAGGCGCTG 174
LOC_Os04g39020.1         ATCCCGGCGGCCACGGCGGAGGACGTCGAGCTCGCGGTGTCGGCGGCGAGGGATGCGTTC 180
                         ********** *************** **    ****** ******** **** *** *

LOC_Os08g32870.1_NF      AAGAGGAACCGGGGCCGCGACTGGGCGCGCGCGCCGGGCGCCGTCCGGGCCAAGTACCTC 234
LOC_Os08g32870.1_F       AAGAGGAACCGGGGCCGCGACTGGGCGCGCGCGCCGGGCGCCGTCCGGGCCAAGTACCTC 234
LOC_Os04g39020.1         GGCCGCGACGGTGGGAGACACTGGTCGCGCGCGCCTGGGGCCGTGCGGGCCAAGTACCTC 240
                             *  ** * **  *  ***** ********** ** ***** ***************

LOC_Os08g32870.1_NF      CGCGCAATCGCGGCCAAGATAATCGAGAGGAAATCTGAGCTGGCTAGACTAGAGACGCTT 294
LOC_Os08g32870.1_F       CGCGCAATCGCGGCCAAGATAATCGAGAGGAAATCTGAGCTGGCTAGACTAGAGACGCTT 294
LOC_Os04g39020.1         AAGGCGATCGCCGCTAAGATTAAAGATAAGAAATCTTATCTAGCTTTGTTGGAGACTCTT 300
                            ** ***** ** ***** *  ** * ******* * ** ***    * ***** ***

LOC_Os08g32870.1_NF      GATTGTGGGAAGCCTCTTGATGAAGCAGCATGGGACATGGACGATGTTGCTGGATGCTTT 354
LOC_Os08g32870.1_F       GATTGTGGGAAGCCTCTTGATGAAGCAGCATGGGACATGGACGATGTTGCTGGATGCTTT 354
LOC_Os04g39020.1         GATTCTGGGAAGCCTCTGGATGAAGCAGCTGGGGACATGGAGGATGTCGCTGCATGCTTT 360
                         **** ************ ***********  ********** ***** **** *******

LOC_Os08g32870.1_NF      GAGTACTTTGCAGATCTTGCAGAATCCTTGGACAAAAGGCAAAATGCACCTGTCTCTCTT 414
LOC_Os08g32870.1_F       GAGTACTTTGCAGATCTTGCAGAATCCTTGGACAAAAGGCAAAATGCACCTGTCTCTCTT 414
LOC_Os04g39020.1         GAGTATTATGCTGATCTGGCAGAAGCTTTAGATGGGAAACAACGGGCACCAATCTCTCTA 420
                         ***** * *** ***** ****** * ** **    *  ***   *****  *******

LOC_Os08g32870.1_NF      CCAATGGAAAACTTTAAATGCTATCTTCGGAAAGAGCCTATCGGTGTAGTTGGGTTGATC 474
LOC_Os08g32870.1_F       CCAATGGAAAACTTTAAATGCTATCTTCGGAAAGAGCCTATCGGTGTAGTTGGGTTGATC 474
LOC_Os04g39020.1         CCCATGGAAAATTTTGAGTCCTATGTACTCAAAGAACCCATTGGGGTTGTTGGACTTATC 480
                         ** ******** *** * * **** * *  ***** ** ** ** ** *****  * ***

LOC_Os08g32870.1_NF      ACACCTTGGAACTATCCTCTCCTGATGGCAACATGGAAGGTAGCTCCTGCCCTGGCTGCT 534
LOC_Os08g32870.1_F       ACACCTTGGAACTATCCTCTCCTGATGGCAACATGGAAGGTAGCTCCTGCCCTGGCTGCT 534
LOC_Os04g39020.1         ACTCCCTGGAATTATCCTCTGCTGATGGCTACTTGGAAGGTTGCACCTGCCCTGGCTGCT 540
                         ** ** ***** ******** ******** ** ******** ** ***************

LOC_Os08g32870.1_NF      GGCTGTACAGCTGTACTAAAACCATCTGAATTGGCTTCCGTGACTTGTTTGGAGCTTGCT 594
LOC_Os08g32870.1_F       GGCTGTACAGCTGTACTAAAACCATCTGAATTGGCTTCCGTGACTTGTTTGGAGCTTGCT 594
LOC_Os04g39020.1         GGGTGTACAGCTGTATTAAAGCCATCTGAGCTTGCTTCCCTGACATGTTTAGAGCTTGGT 600
                         ** ************ **** ********  * ****** **** ***** ******* *

LOC_Os08g32870.1_NF      GATGTGTGTAAAGAGGTTGGTCTTCCTTCAGGTGTGCTAAACATAGTGACTGGATTAGGT 654
LOC_Os08g32870.1_F       GATGTGTGTAAAGAGGTTGGTCTTCCTTCAGGTGTGCTAAACATAGTGACTGGATTAGGT 654
LOC_Os04g39020.1         GGAATATGTGCAGAAATTGGATTACCTCCAGGAGTCTTGAACATAATTACTGGTCTGGGC 660
                         *   * ***  ***  ****  * *** **** **  * ****** * *****  * **

LOC_Os08g32870.1_NF      TCTGAAGCCGGTGCTCCTTTGTCATCACACCCTGGTGTAGACAAGGTTGCATTTACTGGG 714
LOC_Os08g32870.1_F       TCTGAAGCCGGTGCTCCTTTGTCATCACACCCTGGTGTAGACAAGGTTGCATTTACTGGG 714
LOC_Os04g39020.1         ACTGAAGCTGGTGCTCCATTAGCTTCACATCCCCATGTGGATAAGATTGCTTTTACTGGA 720
                          ******* ******** **  * ***** **   *** ** *** **** ********

LOC_Os08g32870.1_NF      AGTTATGAAACTGG---------TATGGCTTCAGCTGCTCCTATGGTTAAGCCTGTTTCA 766
LOC_Os08g32870.1_F       AGTTATGAAACTGGTAAAAAGATTATGGCTTCAGCTGCTCCTATGGTTAAGCCTGTTTCA 774
LOC_Os04g39020.1         AGCACAGAAACTGGTAAGAGGATAATGATTACTGCTTCCCAAATGGTCAAGCCTGTTTCG 780
                         **    ********          ***  * * *** * *  ***** ***********
```

```
LOC_Os08g32870.1_NF      CTGGAACTTGGTGGAAAAAGTCCTATAGTGGTGTTTGATGATGTTGATGTTGAAAAAGCT 825
LOC_Os08g32870.1_F       CTGGAACTTGGTGGAAAAAGTCCTATAGTGGTGTTTGATGATGTTGATGTTGAAAAAGCT 834
LOC_Os04g39020.1         TTAGAGCTTGGTGGCAAAAGTCCTCTTATTGTCTTTGATGATGTTGATATTGATAAAGCT 840
                          * ** ******** ********* *  * ** *************** **** ******

LOC_Os08g32870.1_NF      GTTGAGTGGACTCTCTTTGGTTGCTTTTGGACCAATGGCCAGATTTGCAGTGCAACATCG 885
LOC_Os08g32870.1_F       GTTGAGTGGACTCTCTTTGGTTGCTTTTGGACCAATGGCCAGATTTGCAGTGCAACATCG 894
LOC_Os04g39020.1         GTTGAGTGGGCCATGTTTGGGTGTTTTGCGAACGCTGGTCAAGTCTGCAGTGCTACTTCT 900
                         ********* *  * ***** ** ***  ** *  *** **  * ******** ** **


LOC_Os08g32870.1_NF      CGTCTTATTCTTCATAAAAAAATCGCTAAAGAATTTCAAGAAAGGATGGTTGCATGGGCC 945
LOC_Os08g32870.1_F       CGTCTTATTCTTCATAAAAAAATCGCTAAAGAATTTCAAGAAAGGATGGTTGCATGGGCC 954
LOC_Os04g39020.1         CGTCTACTTTTGCATGAGAAAATTGCAAAGCGATTCTTGGATAGGTTGGTTGCATGGGCA 960
                         *****  ** * *** * ***** ** **   ***    ** *** *************

LOC_Os08g32870.1_NF      AAAAATATTAAGGTGTCAGATCCACTTGAAGAGGGTTGCAGGCTTGGGCCCGTTGTTAGT 1005
LOC_Os08g32870.1_F       AAAAATATTAAGGTGTCAGATCCACTTGAAGAGGGTTGCAGGCTTGGGCCCGTTGTTAGT 1014
LOC_Os04g39020.1         AAGAGTATCAAAATCTCAGATCCACTAGAAGAAGGTTGCAGGCTGGGGTCAGTCGTTAGT 1020
                         ** * *** **  * *********** ***** *********** *** * ** ******

LOC_Os08g32870.1_NF      GAAGGACAGTATGAGAAGATTAAGCAATTTGTATCTACCGCCAAAAGCCAAGGTGCTACC 1065
LOC_Os08g32870.1_F       GAAGGACAGTATGAGAAGATTAAGCAATTTGTATCTACCGCCAAAAGCCAAGGTGCTACC 1074
LOC_Os04g39020.1         GAAGGGCAGTATCAAAAAATAATGAAGTTCATCTCAACAGCAAGATGTGAAGGTGCCACA 1080
                         ***** ****** * ** ** * * * **  * ** ** ** * * *  ******* **

LOC_Os08g32870.1_NF      ATTCTGACTGGTGGGGTTAGACCCAAGCATCTGGAGAAAGGTTTCTATATTGAACCCACA 1125
LOC_Os08g32870.1_F       ATTCTGACTGGTGGGGTTAGACCCAAGCATCTGGAGAAAGGTTTCTATATTGAACCCACA 1134
LOC_Os04g39020.1         ATTCTATATGGGGGTGCCCGACCACAACACCTCAAAAGGGGGTTCTTTATTGAGCCTACT 1140
                         *****   *** ** *   ****  * ** **  * *  ** **** ****** ** **

LOC_Os08g32870.1_NF      ATCATTACTGATGTCGATACATCAATGCAAATTTGGAGGGAAGAAGTTTTTGGTCCAGTG 1185
LOC_Os08g32870.1_F       ATCATTACTGATGTCGATACATCAATGCAAATTTGGAGGGAAGAAGTTTTTGGTCCAGTG 1194
LOC_Os04g39020.1         ATTATAACAAATGTTAGCACATCAATGCAAATTTGGCGAGAGGAAGTCTTTGGACCGGTC 1200
                         ** ** **  ****    ****************** * ** ***** ***** ** **

LOC_Os08g32870.1_NF      CTCTGTGTGAAAGAATTTAGCACTGAAGAAGAAGCCATTGAATTGGCCAACGATACTCAT 1245
LOC_Os08g32870.1_F       CTCTGTGTGAAAGAATTTAGCACTGAAGAAGAAGCCATTGAATTGGCCAACGATACTCAT 1254
LOC_Os04g39020.1         ATCTGCGTTAAAGAATTTAGGACAGAGCGTGAAGCAGTAGAACTTGCAAATGATACTCAC 1260
                          **** ** *********** ** **    *****  * *** * ** ** ********

LOC_Os08g32870.1_NF      TATGGTCTGGCTGGTGCTGTGCTTTCCGGTGACCGCGAGCGATGCCAGAGATTAACTGAG 1305
LOC_Os08g32870.1_F       TATGGTCTGGCTGGTGCTGTGCTTTCCGGTGACCGCGAGCGATGCCAGAGATTAACTGAG 1314
LOC_Os04g39020.1         TATGGTCTAGCTGGCGCTGTGATTTCCAATGATCTAGAGAGGTGCGAGCGCATTTCAAAG 1320
                         ******** ***** ****** *****  *** *  *** * *** ** *  *  *  **

LOC_Os08g32870.1_NF      GAGATCGATGCCGGAATTATCTGGGTGAACTGCTCGCAACCCTGCTTCTGCCAAGCTCCA 1365
LOC_Os08g32870.1_F       GAGATCGATGCCGGAATTATCTGGGTGAACTGCTCGCAACCCTGCTTCTGCCAAGCTCCA 1374
LOC_Os04g39020.1         GCTATCCAGTCAGGTATCGTTTGGATAAATTGCTCGCAACCATGCTTTGTTCAAGCTCCA 1380
                         *  *** *  * ** **  * *** * ** *********** *****    *********

LOC_Os08g32870.1_NF      TGGGGCGGGAACAAGCGCAGCGGCTTTGGACGCGAGCTCGGAGAAGGGGGCATTGACAAC 1425
LOC_Os08g32870.1_F       TGGGGCGGGAACAAGCGCAGCGGCTTTGGACGCGAGCTCGGAGAAGGGGGCATTGACAAC 1434
LOC_Os04g39020.1         TGGGGAGGGAACAAGCGGAGTGGTTTTGGCCGGGAGCTAGGACAGTGGGGCCTCGATAAC 1440
                         ***** *********** ** ** ***** ** ***** *** *  ***** * ** ***

LOC_Os08g32870.1_NF      TACCTAAGCGTCAAGCAAGTGACGGAGTACGCCTCCGATGAGCCGTGGGGATGGTACAAA 1485
LOC_Os08g32870.1_F       TACCTAAGCGTCAAGCAAGTGACGGAGTACGCCTCCGATGAGCCGTGGGGATGGTACAAA 1494
LOC_Os04g39020.1         TACTTGAGCGTGAAGCAAGTCACCAAGTACTGCTCAGATGAACCATACGGATGGTACCGG 1500
                         *** * ***** ******** **  *****  *** ***** ** *  *********

LOC_Os08g32870.1_NF      TCCCCTTCCAAGCTGTAA     1503
LOC_Os08g32870.1_F       TCCCCTTCCAAGCTGTAA     1512
LOC_Os04g39020.1         CCTCCATCCAAGCTGTAG     1518
                          * ** ***********
```
