# Supplementary material for: Creation of novel alleles of fragrance gene OsBADH2 in rice through CRISPR/Cas9 mediated gene editing
Source: PLoS One. 2020 Aug 12;15(8):e0237018. doi: 10.1371/journal.pone.0237018 (PMC7423090; doi:10.1371/journal.pone.0237018)
Supplement: Attachment — Submitted filename: Responses to Reviewers.docx [file pone.0237018.s009.docx]

| **S. No** | **Comments of the Reviewers** | **Replies to Reviewer’s comments** |
| --- | --- | --- |
| **Reviewer # 1** | | |
| 1 | The authors should include the aroma chemistry in the introduction to explain the function of BADH2 protein in detail, and how it may regulate the aroma threshold in rice. | Authors are thankful to the reviewer for the valuable suggestion. We have included a brief note on the aroma chemistry under “Introduction” (Line Nos: 72-76) along with suitable reference (in the revised manuscript with track changes file) |
| 2 | I was wondering if the authors have observed any other effects in rice progenies with edited BADH2? Growth vigor, stress response, etc. | The authors did not observe any notable phenotypic abnormalities in the T_0_ and T_1_ progenies analysed. A detailed agronomic evaluation of edited lines will be carried out in later generations using the homozygous mutants identified. |
| 3 | The authors did not mention other BADH's in rice. Two BADH homologs are present in rice, BADH1, and BADH2, with ~75% sequence similarity between them. BADH1 is associated primarily with abiotic stress tolerance, while BADH2 is known to attribute to rice aroma. The authors should mention such critical aspects related to BADH. | As per the suggestions made by the reviewer, we have added discussion on the homology between BADH1 and BADH2 (Line number 380-386 in the revised manuscript with track changes file). Necessary precautions were taken during the designing of sgRNA to specifically target the BADH2. We have added Supplementary Figure 6 (S6) including the above details |
| 4 | The authors performed comparative profiling of volatile compounds in the T1 edited lines; This is the critical result in context with the study objective. The authors must present the change in the pyrrolidone levels in edited lines compared with the non-transgenic ASD16 line. These results should be in the main text. | Authors are thankful to the reviewer for the critical observation and suggestion. Pyrrolidone and other novel aromatic compounds were detected only in the gene edited lines; These aromatic compounds were not at all detected in the non-transgenic ASD16. We have included a figure (Figure 6) showing the chromatograms of non-transgenic ASD16 and 2 edited lines indicating the peak of aromatic compounds in the edited lines (Line Nos # 315 – 319 in the revised manuscript with track changes file). |
| 5 | The conclusion is very superficial. The authors can improve the conclusion section more precisely | As suggested by the reviewer, conclusion statements have been rewritten with more precision (Line # 442 – 452 in the revised manuscript with track changes file). |
| 6 | In the second paragraph, aro8.1 and badh2 should be in uppercase, also several other places the gene names mentioned in the lowercase; please check | Authors are thankful to the reviewer for the critical observations to improve the quality of the manuscript. We have carried out the suggested changes throughout the manuscript |
| **Reviewer 2** | | |
| 7 | Initial PCR screening shows altered allelic pattern in 10 progenies, while 13 progenies were sequenced to check the mutation. On what basis 13 progenies were selected for sequencing | As mentioned in the manuscript (Lines 220 – 226 in the revised manuscript with track changes file), thirteen progenies producing aroma through sensory evaluation test in T_0_ generation were selected for sequencing. |
| 8 | The Supplementary Figure S2 indicates multiple bands in many progenies, why those lines were not highlighted | As mentioned in the manuscript (Line # 214-218 in the revised manuscript with track changes file) all the 211 T_0_ progenies were subjected to PCR analysis and sensory evaluation test. Thirteen progenies producing aroma in T0 generation were alone selected for sequencing. Further experiments are in progress to advance the other progenies to subsequent generations and study the phenotype. Hence, they were not highlighted. |
| 9 | Justify the variation existing within the progenies of same event for ex. 2-17 & 2-19 and 7-1 & 7-2 | Such variations of mutations existing within the progenies of same event are already reported in genome editing (Estefania et al., 2018 in Poplar;) |
| 10 | Only two lines were forwarded and screened in the T1 generation, whether other progenies with mild aroma were tested in T1 generation. The probability of generating a vector free mutant line can be increased through screening several progenies. | Authors are thankful to the reviewer for the valuable suggestions. In the present study, two progenies producing strong aroma were alone forwarded to T_1_ generation. Efforts will be made to screen other progenies producing mild aroma. We will take the suggestions of the Reviewer to screen large number of mutant progenies to obtain vector free lines. |
| 11 | The frequency of +1/-1 mutation seems to be high, which does not lead to strong phenotype. | High frequency of small/minor mutations are commonly reported in CRISPR/Cas9 mediated genome editing. Similarly, our study also documented high frequency of +1/-1 mutations and these mutations did not lead to aroma. However, we will plan to analyse the progenies in later generations. |
| 12 | English language correct will improve the MS | We have tried to improve the language of the manuscript to the possible extent |
